# Retrospective evaluation of the pediatric multicystic dysplastic kidney patients: experience of two centers from southeastern Turkey

**DOI:** 10.3906/sag-2011-175

**Published:** 2021-06-28

**Authors:** Mehtap AKBALIK KARA, Aysel TAKTAK, Caner ALPARSLAN

**Affiliations:** 1 Department of Pediatric Nephrology, Faculty of Medicine, Gaziantep University, Gaziantep Turkey; 2 Department of Pediatric Nephrology, Faculty of Medicine, Mustafa Kemal University, Antakya Turkey; 3 Department of Pediatric Nephrology, İzmir Tepecik Training and Research Hospital, İzmir Turkey

**Keywords:** Multicystic dysplastic kidney, children, vesicoureteral reflux, contralateral anomalies

## Abstract

**Background/aim:**

The objective of this study is to determine the clinical features of unilateral multicystic dysplastic kidney (MCDK) patients.

**Materials and methods:**

The demographic, clinical, laboratory, and radiologic features of MCDK patients at Diyarbakır Children’s Hospital and Diyarbakır Gazi Yaşargil Training and Research Hospital between January 2008-June 2019 were retrospectively evaluated.

**Results:**

A total of 111 [59 (53.2%) male and 52(46.8%) female] patients with MCDK were followed for a mean period of 41.89 ± 32.03 months. MCDK was located on the left and right sides in 46 (41.4%) and 65 (58.6%) of the children, respectively (p > 0.05). A total of 87 (78.4%) patients had antenatal diagnosis. The mean age at diagnosis was 13.7 ± 34.2 months. Of the 49 voiding cystourethrogram (VCUG)-performed patients, vesicoureteral reflux was detected in 11 patients (22.4%). Other associated urological anomalies in the patients were detected in 12 (10.8%) patients. On Tc-99m dimercaptosuccinic acid (DMSA) scintigraphy which was performed in all patients showed scarring in four children. Eight patients had history of UTI (7.2%). Renal failure, hypertension, and proteinuria were diagnosed in three children (2.7%). Sixty-nine (62%) patients developed compensatory hypertrophy.

**Conclusion:**

All cases should be followed up closely and VCUG should be reserved for patients with recurrent UTI and other urological problems indicated by ultrasonography and abnormal DMSA scan results.

## 1. Introduction

Multicystic dysplastic kidney (MCDK) is a developmental anomaly of the kidney and it is the most common cause of cystic renal disease in children [1]. It is characterized by the enlargement of the kidney, multiple, noncommunicating cysts of varying size, no functioning kidney parenchyma, and atresia or hypoplasia of the ureter [2]. The incidence is 1 in 1000–4300 live births [3]. Although the use of antenatal and postnatal ultrasonography (US) has become wide spread in the past 20 years, MCDK may still be asymptomatic and may not be detected until adulthood [4]. The condition occurs more commonly in males than females (2.4:1) and the left kidney is more affected than the right kidney [5]. MCDK is mostly an isolated condition; however, the contralateral urinary tract could be affected by other abnormalities such as vesicoureteral reflux (VUR), ureteropelvic junction obstruction (UPJO) and ureterovesical obstruction (UVJO) [6,7].

There are three major issues in patients with unilateral MCDK and the fate of the contralateral functioning kidney [8]. Firstly, MCDK may undergo complete or partial involution within time [9]. Secondly, although it is presumably thought to have increased risk for the incidence of Wilms tumor in the past, it is not widely accepted today [10]. And thirdly, the risk of hypertension in MCDK has been found to be high but no higher than in the general pediatric population [8].

Although the US is the first-line and basic imaging technique for the follow-up period of MCDK, renal scintigraphy provides additional information such as the function of renal cortical tissue in MCDK, the involvement of contralateral kidney, and/or presence of any scar and/or hypodysplasia. Therefore, it is suggested to perform the Tc-99m dimercaptosuccinic acid (DMSA) scintigraphy at least once at a follow-up period in MCDK patients [10–13].

 The management of MCDK has changed greatly over time. Previously, nephrectomy was performed to avoid infection, pain, hypertension, and malignancy [14]. Recently, management is primarily conservative due to favorable outcome of patients [15].

Here we present 11-year of retrospective data from 111 MCDK patients. We primarily wanted to describe the clinical, laboratory findings and secondarily, we wanted to discuss whether routine VCUG should be performed with no history of urinary tract infections and no pathological findings on US and DMSA scintigraphy imaging. 

## 2. Materials and methods

### 2.1. Study design

We retrospectively reviewed the medical records of 139 patients with MCDK whom were followed at Diyarbakır Children’s Hospital and Diyarbakır Gazi Yaşargil Training and Research Hospital from January 2008 to June 2019. As we excluded 28 patients who had incomplete medical records and irregular follow-up or whose follow-up duration time was less than 1 year, who had other genetic and chromosome abnormalities, spinal cord diseases, or anal atresia. A total of 111 patients were enrolled in this study. We evaluated clinical parameters including sex, age at diagnosis, parental consanguinity, family history for renal diseases, side of the affected kidney, renal function tests (serum urea and creatinine levels), estimated glomerular filtration rate (e-GFR) with Schwartz Formula [16], history of urinary tract infection (UTI), hypertension, malignancy, additional urological anomalies, congenital heart diseases, previous operations, nephrectomy indications for those that underwent nephrectomy because of MCDK and finally follow-up time. 

### 2.2. Diagnosis and follow-up

The diagnosis of MCDK was made according to ultrasonography. The diagnosis was based on the following established ultrasound criteria: (1) multiple cysts of varying size, (2) absence of normal renal sinus echoes, and (3) absence of normal renal parenchyma [17]. The absence of Tc-99m dimercaptosuccinic acid (DMSA) scintigraphy was used to confirm the diagnosis of MCDK. Compensatory hypertrophy of the contralateral kidney was defined as renal length >2 standard deviations (SD) of the mean value of normal kidneys according to age [18].

 Patients with neither UTI nor abnormality of contralateral kidney were assessed with urinalysis, urinary culture, and control US of urinary system every 3 months until 1 year of age. The same evaluation was performed every 6 months between 1 and 2 years of age and annually or twice per year thereafter. Those with a history of UTI and abnormality of contralateral kidney were evaluated according to the condition of the patients. The urea and creatinine levels were checked in the first week of life for the patients who were diagnosed antenatally and they were checked out once per year or year after. Hypertension is defined as each systolic and/or diastolic blood pressure ≥95th percentile measured on three or more separate occasions for age, sex, and height [19].

### 2.3. Performing voiding cystourethrogram (VCUG) and evaluation of urinary tract infection

VCUG was performed in the patients who had US abnormalities in the contralateral kidney (e.g., hydronephrosis or hydroureteronephrosis, increased echogenicity in the renal parenchyma), scan abnormality on the unaffected kidney and/or UTI. Vesicoureteral reflux on VCUG was graded according to the international classification for grading of VUR [20]. Antibiotic prophylaxis was given to patients with high grades of VUR and continued till resolution or decline of VUR grade or completing of toilet training. If patients had suspected of UTI, with symptoms such as fever, oral intolerance in infancy, dysuria, vomiting, abdominal pain, and frequency, urine samples were collected. Urinary tract infection was defined as the presence of pyuria (≥5 white blood cells per high-power field), and/or positive leukocyte esterase test or nitrite, and a result of a positive urine culture test. Any growth of a single bacterium from a suprapubic bladder aspiration, or growth of a single microorganism from ≥105 colony-forming units/mL from a midstream clean-void urine specimen in toilet-trained children, or ≥5 × 104 colony-forming units/mL collected from transurethral catheterized specimen was considered significant.

### 2.4. Evaluation of proteinuria and renal functions

Proteinuria was tested with a urine dipstick method at every outpatient visit, if proteinuria was detected for more than two occasions with the dipstick method, further evaluation was made with spot urine protein/creatinine ratio or 24-h urine collection. Pathological proteinuria was defined as >0.2 mg/mg or >4 mg/m2/h, respectively [21]. Blood urea and creatinine levels and eGFR rates were used to determine renal functions. Chronic kidney disease (CKD) was defined as eGFR <60 mL/min/1.73 m2 for more than 3 months; patients with CKD were further categorized into five groups according to their eGFR and KDOQI guideline [22].

### 2.5. Statistical analysis

SPSS v. 22.0 software (IBM Corp., Armonk, NY, USA) was used for data analysis. The Kolmogorov–Smirnov test was used for evaluating data distribution. Descriptive statistics were presented as number of observations and percentage (%). Parametric data was expressed as mean ± standard deviation (SD) and nonparametric data as median (minimum–maximum). Student’t t-test was used to compare the normally distributed and the Mann–Whitney U test to compare non-normally distributed data. Differences in proportions were assessed using the chi-squared test. The results with p < 0.05 were considered statistically significant.

## 3. Results

The clinical characteristics of 111 patients with MCDK in the study are summarized in Table 1. Of the patients, 59 (53.2%) were male and 52 (46.8%) were female. The mean age at diagnosis was 13.7 ± 34.2 months (range: 1–192 months). A total of 87 (78.4%) patients had antenatal diagnosis. The remaining 24 (21.6%) patients were diagnosed incidentally because of nonspecific abdominal pain and/or UTI. Unilateral MCDK was on the right side in 65 patients (58.6%) and on the left side in 46 patients (41.4%). Follow-up duration was 12–132 months (mean: 41.89 ± 32.03 months) and mean age at last control visits was 4.58 ± 3.89 years (1–17 years). In our study, parental consanguinity and family history of congenital anomalies of the kidney and urinary tract (CAKUT) was detected in 64.9% and 11.7% of the patients, respectively. In these patients, families’ congenital anomalies of kidney and urinary tract anomalies such as renal agenesis, MCDK, renal hypoplasia, and horse kidney were identified.

**Table 1:** Characteristics of children with unilateral multicystic dysplastic kidney.

Feature	Number	Rate (%)
SexFemale/male	52/59	46.8/53.2
Side of multicystic dysplastic kidneyRight/left	65/46	58.6/51.4
Parental consanguinity	72	64.9
Prenatal diagnosis	87	78.4
Family history of renal disease	13	11.7
Decreased renal function	3	2.7
Congenital heart disease	4	3.6
Hypertension	3	2.7
Proteinuria	3	2.7
History of urinary tract infection	8	7.2

Mean serum creatinine level was 0.51 ± 0.23 mg/dL (range: 0.1–0.7 mg/dL) and serum urea level was 23.9 ± 16.2 mg/dL (range: 8–140 mg/dL). The mean eGFR was 153.23 ± 27.52 mL/min/1.73 m2 (range: 23–204 mL/min/1.73m2); unfortunately, three of the patients had renal failure (stage III CKD). Hypertension and proteinuria was diagnosed in three children (2.7%). These patients were also diagnosed with stage III chronic kidney disease.

A total of 49 VCUGs were performed (44.1%) and VUR was detected only in 11 patients (22.4%). In most of the patients, UTI and/or confirmed ureter dilatation was the key point for performing VCUG. Contralateral VUR was detected in 9 patients (18.2%) (Grade I, 2 patients; Grade II, 2 patients; Grade IV, 3 patients; and Grade V, 2 patients) and two patients had bilateral VUR (4.2%) (Table 2). DMSA was performed in all patients. All of them showed no parenchymal function at the affected site. Four children had scarring on DMSA imaging. Three of them had stage III chronic kidney disease. One of these patients had a history of stage I VUR on the affected side of kidney and stage V VUR on the unaffected site, and a history of performing surgical correction and ureteroneocystostomy operation for the unaffected kidney. The other two patients did not have VUR but had hypoplasic and/or dysplasic kidney with neither extrarenal nor chromosomal abnormality. The other patient was a 10-year-old girl who had a history of UTI but had not been evaluated by a pediatric nephrologist. VCUG was performed but VUR was not detected; unfortunately, DMSA revealed scar in the contralateral kidney. There was no statistically significant relationship between renal scar and reflux in our patients (p > 0.999, Fisher’s exact test). Similarly, there was no statistically significant relationship between patients with vesicoureteral reflux and those without vesicoureteral reflux in terms of sex, age at diagnosis, affected side of kidney, follow-up duration, hypertension, proteinuria, urea, creatinine level, and eGFR (p = 0.732, Fisher’s exact test; p = 0.859, Mann–Whitney U; p > 0.999, Fisher’s exact test; p = 0.02, Student’s t-test; p > 0.999, Fisher’s exact test; p > 0.999, Fisher’s exact test; p = 0.764, Mann–Whitney U; p = 0.29, Mann–Whitney U; p = 0.69, Mann–Whitney U). 

**Table 2:** Contralateral vesicoureteral reflux and other urinary system anomalies accompanying to multicystic dysplastic kidney.

	Patient number	Rate (%)
Contralateral VUR	9	18.2
Hypdysplasia/dysplasia	2	1.8
UPJO	5	4.5
UVJO	1	0.9
Ipsilateral ureteroceles	3	2.7
Undescended testis	2	1.8
Hypospadias	1	0.9

VUR: Vesicoureteral reflux, UPJO: Ureteropelvic junction obstruction, UVJO: Ureterovesical junction obstruction.

Of the 111 patients, 4 children had undergone nephrectomy in other centers. In our clinic, none of the remaining patients underwent surgery in the follow-up duration. We were not able to evaluate the involution rates and the reason of nephrectomy because of incomplete data. On the other hand, compensatory hypertrophy in the contralateral kidney was detected in 69 (62%) of the patients. No malignant transformation was observed in patients.

The other associated urologic anomalies are given in Table 2. While surgical procedure was performed in patients with hypospadias and undescended testicles, UPJO and UVJO patients did not require any operation. Congenital heart disease was the most common extrarenal finding. Of the 4 patients (3.6%), 3 had atrial septal defect and 1 had ventricular septal defect. One patient had mental retardation and epilepsy due to hypoxic birth history.

## 4. Discussion

The frequency of diagnosis of MCDK has increased in recent years because of the widespread use of antenatal and postnatal ultrasound [23,24]. MCDK is accepted to be a benign condition but early identification of associated abnormalities and appropriate management of these patients are crucial in children due to having solitary kidney [25–27]. 

In the present series, unilateral MCDK was seen more commonly in males than in females, and mostly the left kidney was more affected than the right [5]. The study includes 111 children with a male-to-female ratio of 1.13. Our study revealed that males are more affected than females but right side involvement was higher apart from the literature. In the literature, prenatal diagnosis rate varies related to local features of the institutions. Schreuder et al. reported the prenatal diagnosis rate as 90.8% in their metaanalysis and Moralıoğlu et al. reported the prenatal diagnosis rate as 94.1% of the patients with MCDK [25,26]. Despite these two high rates, Kara et al. reported the diagnosis rate as 50% [11]. In our opinion, the rate may be related with the socioeconomic status of families and regular antenatal follow-up. Our study revealed that prenatal diagnosis rate was 78.4%. In our study, we detected high rates of consanguinity (64.9%), so this situation seems to relate with common consanguineous marriage in our country especially in our region. We found that 11.7% of our patients had CAKUT (renal agenesis, renal hypoplasia, MCDK, horseshoe kidney) in their family members. In a study, 94 family members of MCDK patients for CAKUT were screened, and 3 MCDK and one renal agenesis were detected [28]. Thus, the authors advocated that no formal familial screening recommended and MCDK defined as a sporadic condition. We did not routinely screen our patients’ family members either.

Renal ultrasonography is recommended as a preliminary diagnostic imaging study. According to the DMSA results of a seven-cohort study, among 27 of the 347 patients (7.7%), activity range on DMSA was 1%–18% at the affected site [25]. In the present study, DMSA scan showed no parenchymal function at the affected site in all patients and four children had scar at the unaffected kidney.

Prognosis of patients with MCDK is generally related to the function of the contralateral kidney, and abnormalities of contralateral urinary tract and bladder. Contralateral urological abnormalities were detected in 16 (14.4%) patients in our series. Previous series reported contralateral anomalies in 14.7%–20% of the patients [9,25]. Our study showed quite similar results with these reports. The rate of VUR of the contralateral kidney had been noted between 4.5% and 43.0% in previous studies [26,29,30]. It was 18.2% in our study. Some authors revealed that the rate of the high-grade VUR (grade III, IV, V) in the affected contralateral kidney varied from 26.0% to 50.0% and only a few patients required antireflux surgery [9,25,29]. In our study, only five patients had high-grade reflux to the contralateral kidney and through it only two patients required antireflux surgery. Although two of these patients had a renal scar in the contralateral kidney, the renal scar was already present in the first scintigraphies. In the light of these results, we also thought that routine VCUG was unnecessary like these authors pointed out [29,31]. Since VCUG is an invasive process, it is stressful for both patients and their families, along with exposing radiation, urethral trauma, and iatrogenic UTI [32]. Moreover, a spontaneous resolution is possible, especially in low-grade VUR [33]. Blachman-Braun et al. evaluated 156 MCDK patients; 38 of them had abnormal US findings in the contralateral kidney, and when a VCUG was performed, they found 25 patients with VUR; however; only two of them had severe VUR. They showed a statistical correlation between the severity of VUR and abnormal US findings [34]. Therefore, they concluded that patients with abnormal contralateral US findings would benefit the most from VCUG. However, despite the evidence supporting an association between UTI and VUR, there is no convincing evidence that either UTI or VUR contributes to the progression of CKD to end-stage kidney disease (ESKD); in these patients, the main cause of CKD/ESKD could be the presence of the hypoplastic kidney itself rather than the VUR [35]. Although US findings could predict patients with severe VUR, evaluation of patients for UTI with a DMSA scan would provide a more effective estimation. 

There are some other urological problems including CAKUT that could be detected in MCDK. Contralateral UPJO rates vary between 1.1% and 13% in previous studies [25,26]. Our study also indicated that UPJO rates were 4.5%, and none of these patients required surgery. Ipsilateral urological abnormalities were obtained in three of our patients (2.7%). All were ipsilateral nonsymptomatic ureteroceles. In a report, the authors detected ipsilateral ureteroceles in six patients (4.9%) [7]. Thus, our coexisting CAKUT spectrum anomalies are comparable with the literature.

Multicystic dysplastic kidney is generally asymptomatic. Abdominal or flank pain and respiratory distress are uncommon symptoms because of the pressure effect of the abnormal kidney [11]. In our cohort, all patients were asymptomatic. The complications of MCDK include hypertension, UTI, and renal malignancy. One of the important complications of MCDK is UTI and it has been reported in 5%–34.7% of the patients [25,29]. In a study by Calaway et al., no statistically significant relationship was found between UTI and renal scar, with or without VUR [10]. We also reached similar results in our study. 

The rate of hypertension in MCDK is not well-defined and has wide published rates from 0.6% to 17.7% [14]. A nonfunctional bloodless kidney could be the cause of hypertension in infancy and early childhood. Indeed, presence of any accompanying congenital urinary abnormality such as UPJO, renal dysplasia, and development of a pyelonephritic scar secondary to VUR and the effects of hyperfiltration over time in the contralateral kidney has been considered a potential cause of hypertension [11]. In the present study, hypertension was diagnosed in three patients (2.7%). One of the patients had high-grade reflux to contralateral kidney with renal scarring. The other two patients had contralateral renal dysplasia. Both of these patients were considered to have stage III CKD, and antihypertensive treatment was initiated.

Contralateral kidney usually undergoes compensatory hypertrophy. The rate of compensatory hypertrophy of the contralateral kidney varies from 16.6% to 89.8%. [7,26,29]. It is considered that if there is no hypertrophy, the practitioner should be suspicious of the contralateral kidney having an abnormality [9]. We detected compensatory hypertrophy in 69 patients (62%); therefore, except three CKD patients, the rest of the study group had normal kidney functions and dimensions. Our result was 62% (69 patients) and was in line with the literature. It may be possible to observe compensatory hypertrophy during longer follow-up. It is well known that kidneys with MCDK involute within time, but there are variable data about the time of involution in the literature [3]. Unfortunately, we were not able to give our involution data because of missing files.

It is already known that MCDK often undergoes involution and malignant transformation has been reported very rare and strict blood pressure measurement and US examination for hypertension are recommended [36–38]. During the follow-up period of our patients, no patient was suspected of malignant transformation. In our follow-up, nephrectomy has not been performed in any case, while four patients underwent nephrectomy in other centers.

Our study is with limitations such as retrospective methodology, a retrospective evaluation of the frequency and characteristics of the UTI, no records of prophylactic antibiotics, and ultrasonographies performed by different radiologists.

## 5. Conclusion

Multicystic dysplastic kidney is the most common cause of renal cystic disease with a good prognosis in childhood. We advocate that VCUG should be reserved for patients with recurrent UTI and other urological problems indicated by US and abnormal DMSA scan results. These decrease unnecessary VSUG imaging. Moreover, pediatric nephrologists should be careful about that hypertension, renal dysfunction, proteinuria, and malignant transformation could develop in a small part of the MCDK patients.

## Ethical approval

The procedures were in accordance with the ethical standard for human experimentations established by Declaration of Helsinki 1975, as revised in 2013. The study was approved by the Ethics Committee of (2018/180) Diyarbakır Gazi Yaşargil Training and Research Hospital.
